# Amorphous Framework in Electrodeposited CuBiTe Thermoelectric Thin Films with High Room-Temperature Performance

**DOI:** 10.1021/acsaelm.1c00063

**Published:** 2021-04-07

**Authors:** N. Padmanathan, Swatchith Lal, Devendraprakash Gautam, Kafil M. Razeeb

**Affiliations:** † Micro-Nano Systems Centre, Tyndall National Institute, University College Cork, Dyke Parade, Lee Maltings, Cork T12 R5CP, Ireland; ‡ Department of Physics, Karpagam Academy of Higher Education, Coimbatore, Tamilnadu 641021, India

**Keywords:** thermoelectrics, electroplating, codeposition, ternary alloy, nano-precipitates, amorphous thin films, power factor

## Abstract

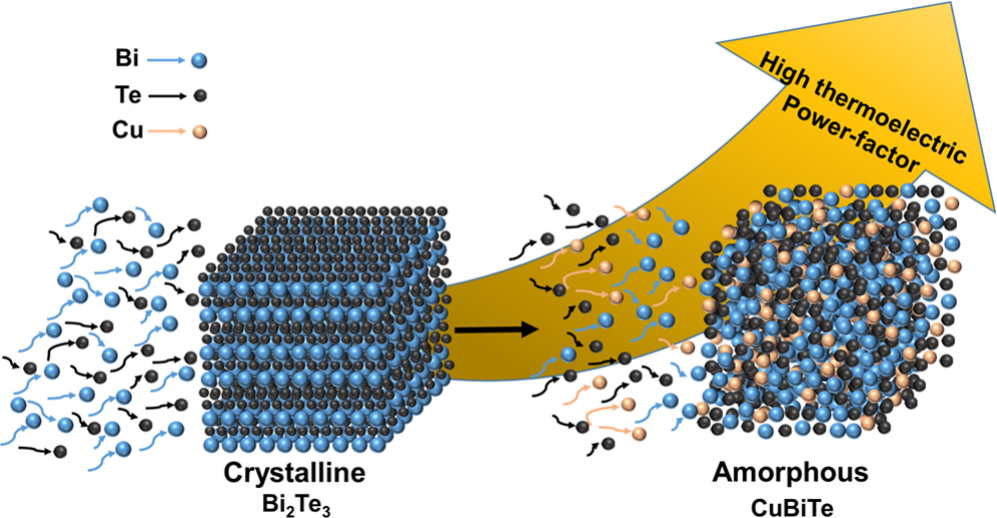

Bismuth telluride-based alloys are the most efficient thermoelectric materials near room temperature and widely used in commercial thermoelectric devices. Nevertheless, their thermoelectric performance needs to be improved further for wide-scale implementation either as a thermoelectric generator or cooler. Here, we propose a simultaneous codeposition of CuBiTe thin films and their phase transition strategy via the traditional electrodeposition process. With just 13 atom % Cu doping, crystalline-to-amorphous phase transformation resulted for the electroplated CuBiTe alloy. A close look at the alloy composition revealed spike-shaped nanocrystalline Bi_2_Te_3_ embedded in the CuBiTe amorphous matrix. Our result shows an exceptionally high power factor (3.02 mW m^–1^ K^–2^), which comes from the enhanced Seebeck coefficient (−275 μV K^–1^) and high electrical conductivity (3.99 × 10^4^ S m^–1^) of CuBiTe films. Therefore, it can be suggested that the adopted strategy to form a unique nanocrystallite-embedded amorphous framework provides a platform to develop next-generation high-performance thermoelectric materials with an extraordinary power factor.

## 1 Introduction

The recent advancements in wireless sensor networks (WSNs) have the potential to realize the “Internet of Things” (IoT), aiming to integrate the physical world with the computer-based systems. These versatile WSNs have been widely used in industrial communications,^1^ remote healthcare,^2^ automotive monitoring,^3^ surveillance,^4^ etc. and are a potential candidate to find place in hitherto unexplored applications. However, one of the major impediments in the path of materializing this type of smart connected environment is the perpetual powering of such billions of deployed sensor nodes. Batteries as the only power source not only add unnecessary volume and weight to such miniaturized systems but also need recharging or replacement once their energy depletes. A lot of new strategies have been adopted to enhance the battery performance; however, the current battery technologies are not able to meet the requisites of advanced microelectronic technologies. In this predicament, the most attractive alternative is to scavenge energy from omnipresent ambient energy sources and assisting the energy storage devices for ultimately powering the microelectronic systems.^5,6^

A thermoelectric (TE) generator is well-suited in extending the life of the battery and eventually replaces the battery of these wireless sensors if there is a large temperature differential. Conversely, these thermoelectric devices can be used in niche cooling applications, for example, to maintain stable temperatures in lasers and optical detectors.^7,8^ Therefore, thermoelectric (TE) devices entail highly efficient thermoelectric materials and in-depth understanding of their electron and phonon transport phenomena. The performance of a thermoelectric material is determined by its figure of merit, *zT* = *S*^2^σ*T*/*k*, where *S* is the Seebeck coefficient, σ is the electrical conductivity, *k* is the thermal conductivity, and *T* is the absolute temperature.^7^ Usually, two strategies are followed to improve the *zT* of the thermoelectric materials. First is to find new materials with ultralow thermal conductivity, and the second is the development of low-dimensional systems for an improved power factor (PF = *S*^2^σ).^9−11^ The primary focus of this work is to enhance the power factor of a material by tailoring the internal microstructure using the doping process.

Until now, Bi_2_Te_3_-based alloys have been demonstrated as the best thermoelectric materials near room temperature,^12−15^ whose *zT* values reach greater than unity for both n-type and p-type conduction.^15,16^ To utilize these materials for widespread applications of a thermoelectric device, a minimum *zT* of 4 should be achieved, which remains a formidable challenge.^16^ The problem is that the three parameters in *zT* (*S*, σ, and *k*) are interdependent.^16^ However, these interdependent transport parameters (Seebeck coefficient and electrical conductivity) can be optimized through chemical doping or alloying the crystal structure by tuning the mobility, effective mass, and concentration of the charge carriers through modification of the electronic band structure near the Fermi level, whereas the thermal conductivity (*k* = *k*_e_ + *k*_L_), specifically the lattice thermal conductivity (*k*_L_), can be reduced through phonon scattering at the interfaces by forming multiscale defects and nanostructuring.^17−19^

To date, many of these strategies are adopted to enhance the *zT* by tuning the Bi_2_Te_3_ structure through alloying,^20−22^ superlattice formation,^23^ varying the composition or defect levels,^24−28^ and by designing a hybrid architecture nanocomposite, which facilitate the simultaneous optimization of the electrical and the thermal transport properties.^29^ Recently, enhancement of power factor (*S*^2^σ) was observed for TE materials (e.g., Bi_2_Te_3_, GeTe, PbTe, SnTe, etc.) by introducing an energy barrier in the layered structure. This was mainly due to the carrier filtering effect by introducing a conducting nanophase at the interface to act as phase boundaries.^21^ The nanophases that formed in the thin films are believed to be the effect of the metal-induced crystallization of the dopant metals.^30^ Although control over the nanophases and their transport properties is still challenging, it can be achieved through a desirable choice of dopant and an efficient method for thin film deposition. Among the various methods reported earlier, including atomic layer deposition,^31^ molecular beam epitaxy,^32,33^ arc-melting,^34^ etc., electrodeposition is one of the most versatile fabrication methods owing to its simple operation at low temperature, low cost, and high deposition rate with flexibility to design a material with tunable properties.^35^ Following the traditional electrodeposition process, the simultaneous codeposition greatly influences the structure and composition of metallic alloys. So far, two kinds of codeposition mechanisms have been reported, that is, normal and anomalous, to form metallic alloys.^36^

The electrical and thermal properties of Bi_2_Te_3_ thin films mainly depend on their compositions and stoichiometry. Alternatively, these fundamental thermoelectric features can be tuned by adding different amounts of extra atoms in the Bi_2_Te_3_ crystal structure. The extra atoms play a key role and should be codeposited stably with Bi and Te to control their composition and crystallinity; therefore, it is important to select the appropriate element. Because of its small electrode potential difference (30–50 mV) from Bi, the Cu atom can be codeposited over a wide range of potential/current density without depolarization effect [Abner Brenner, Thesis].^37,38^ Until now, the effect of Cu doping in Bi_2_Te_3_/Bi_2_Se_3_ has been reported by different groups through Cu intercalation, Cu electrodeposition, etc., which has shown enhancement in the thermoelectric properties.^30,38−40^ Recently, Chen et al. have found that the formation of Cu clusters is due to migration from quintuple layers with aging.^41^ Burton et al. showed extremely high thermoelectric performance (*S* = −390 μV K^–1^) in electrochemically copper-doped bismuth tellurium selenide thin films.^42^ The general hypothesis accepted for Cu occupation in Bi_2_Te_3_ is the replacement of Bi position. In reality, there are unusual structural changes also been caused by Cu addition in Bi_2_Te_3_ such as crystal symmetry disorder, phase transformation, change in microstructure, and elemental composition. Nevertheless, the qualitative structural analysis of Cu occupation in the layered structure is not yet reported, in particular, for thin films. Meanwhile, few amorphous metallic alloy-based thermoelectric materials have also been reported in the literature,^43,44^ and recently Cu ion liquid-like thermoelectrics have received considerable attention.^45^ However, most of these reported materials are bulk powder-sintered materials that show better thermoelectric performance at high temperatures (*zT* = 1.5@1000 °C).^46^ Therefore, a truly high-performance material working at near room temperature and that can be fabricated using silicon-fab-compatible techniques for volume production remains unexplored.

In this work, we have evaluated the room-temperature phase transformation and microstructural changes of CuBiTe films with Cu addition by the simultaneous electro-codeposition technique. The detailed structural changes and the thermoelectric properties of CuBiTe films are systematically investigated for different Cu concentrations. In light of the structural and thermoelectric data, we evaluate the possible occupation of Cu in the CuBiTe ternary alloy thin films. Finally, we propose an electrochemically deposited CuBiTe ternary alloy with embedded Bi_2_Te_3_ nanocrystals as a potential thermoelectric material with the highest power factor of 3.02 mW m^–1^ K^–2^ for room-temperature applications.

## 2 Experimental Section

### 2.1 Electrodeposition of CuBiTe

The electrodeposition mechanism of CuBiTe is investigated using cyclic voltammetry (CV). The CV experiments are conducted in a conventional three-electrode cell with a CHI660C potentiostat. The reference electrode is a Ag/AgCl/KCl (3M) electrode, the counter electrode is a pure graphite plate electrode, with the Si/SiO_2_ (∼1 μm)/Ti/Au(10/20 nm) substrate (32 × 32 mm^2^) as the working electrode. The substrates are cleaned with deionized (DI) water and then dried under flowing N_2_. The electrolytes are prepared by dissolving adequate quantities of the precursors to give CuCl_2_ (0, 0.5, 1, 1.5, 2, and 4 mM), Bi(NO_3_)_3_ (2 mM), TeO_2_ (4 mM), HNO_3_ (1 M), and NH_4_Cl (0.5 M) as a stabilizing agent. The cyclic voltammograms are recorded at a sweep rate of 10 mV s^–1^, with the potential scanned first in the negative (cathodic) direction. All of the films are electrodeposited at a constant potential of −0.050 V for 2 h at room temperature. After electrodeposition, the substrate is removed from the electrolyte, rinsed with DI water, and dried under flowing N_2_.

### 2.2 Material Characterization

The phase structure in CuBiTe thin films is characterized using the X-ray diffraction (XRD; Philips PW3710-MPD diffractometer) technique with the Cu Kα radiation (λ = 1.54 Å). The sample morphologies are examined by a field emission scanning electron microscope (FEI QUANTA 650 HRSEM) with an attached energy-dispersive X-ray spectrometer (EDX; Oxford Instruments INCA energy system). For transmission electron microscopy (TEM) analysis, a thin film lamella of 600 nm width and 40–50 nm thickness is cut using a focused ion beam (FIB) and fixed on the Mo grid. The microstructure analysis is performed on a transmission electron microscope (JEOL HRTEM-2100) at 200 kV. The Raman spectra are recorded using a Horiba LabRAM HR Evolution Raman spectrometer via a confocal Raman microscope at 632.8 nm excitation. The surface and depth profiles of X-ray photoelectron spectra (XPS) are measured using a Kratos Ultra DLD spectrometer with the Al K_α_ radiation (1486.6 eV). The carbon 1s peak is used as a reference to calibrate the binding energies of the other core-level spectra. The electrical resistivity of the samples is measured by a direct current (DC)-current four-point probe method using Jandel RM-3000, while the Seebeck coefficient is determined using a laboratory-built system from the slope of the thermovoltage versus temperature gradient. Further information on the Seebeck coefficient measurements is given in the Supporting Information (SI). The in-plane electrical conductivity, the carrier concentration (*n*), and the Hall mobility (μ_H_) are measured at room temperature using four-probe van der Pauw geometry and 10 × 10 mm^2^ samples. For Hall measurements, a 1.7 T AC magnetic field is applied using Lake Shore’s fully integrated Hall measurement systems (HMS) (Lake Shore 8400).

## 3 Results and Discussion

### 3.1 Cyclic Voltammetry

**Figure 1:**
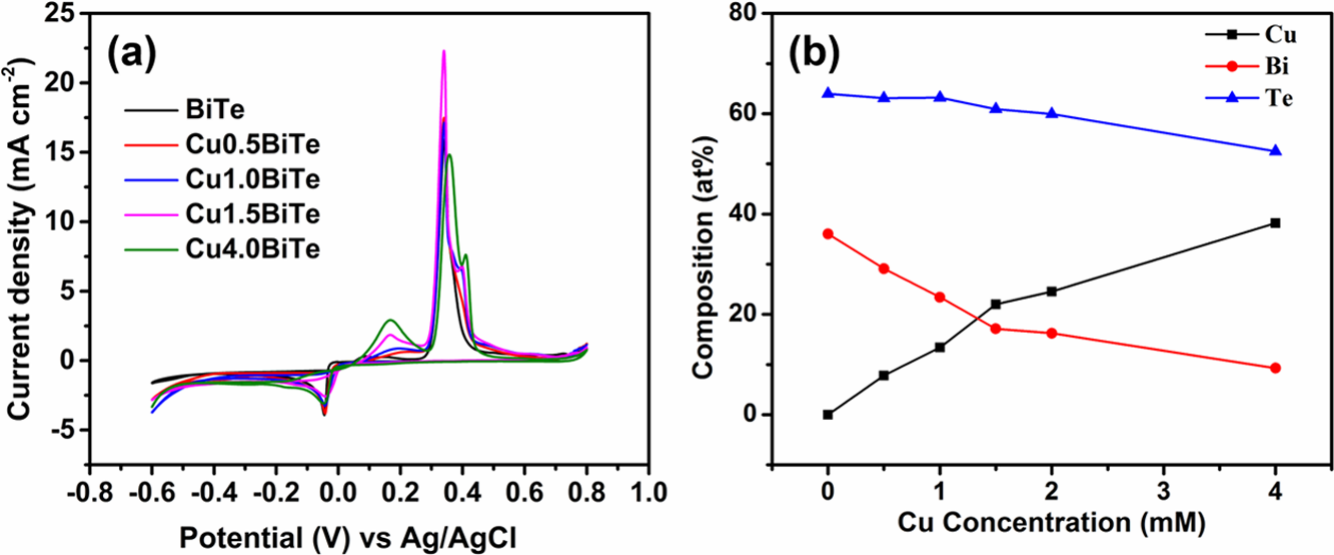
(a) Cyclic voltammograms of BiTe and Cu-added BiTe bath measured at 10 mV s^–1^ and (b) elemental composition of electrodeposited thin films with respect to Cu concentration in the bath solution.

Typical cyclic voltammograms (CVs) of BiTe and CuBiTe are shown in Figure 1a, which are acquired using a standard Au working electrode of 1 mm radius and with a scan rate of 10 mV s^–1^. For a clear observation, the separated CV graphs are shown in Figure S2 (Supporting Information). For the binary Bi–Te solution containing 2 mM Bi(NO_3_)_3_, 4 mM TeO_2_, 1 M HNO_3_, and 0.5 M NH_4_Cl, the CV shows only one reduction peak at −0.045 V, which involves the codeposition of BiTe compounds on the substrate. When the potential sweeps in the positive direction, two anodic peaks are visible between 0 and 0.2 and at 0.340 V, which may correspond to the stripping of Bi(III) and Te(IV) from the BiTe compound. The simultaneous reduction reaction of both Bi and Te is expressed by the following chemical reaction as reported earlier:^35^
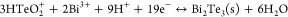
1The CV of the ternary Cu–Bi–Te system, which contains an appropriate quantity of CuCl_2_ (0.5 mM, 1.0 mM, 1.5 mM, and 4 mM) in addition to the fixed Bi(NO_3_)_3_ (2 mM), TeO_2_ (4 mM), HNO_3_ (1 M), and NH_4_Cl (0.5 M), exhibits some distinct features from that of the binary BiTe. On anodic sweeps, three peaks were visible at 0.2, 0.340, and 0.4 V and can be assigned to the oxidation of Bi(0) to Bi(III), Te(0) to Te(IV), and Cu(0) to Cu(II), respectively. Due to the complex electrolyte system, it is difficult to propose the actual chemical reaction for the ternary CuBiTe formation, and it is assumed as follows^39^
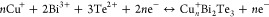
2During the negative sweep, considerable changes have been observed for binary and ternary alloys. In the Bi–Te mixed solution, current density starts to increase from −0.030 V and then reaches the diffusion-limiting current region at −0.045 V. For the ternary system, the deposition potential shifts in the more positive direction than for the binary system. As shown in Figure 1a, the codeposition of CuBiTe starts at −0.016, −0.006, −0.005, and −0.013 V for 0.5, 1.0, 1.5, and 4 mM CuCl_2_ mixed solution baths, respectively. This confirms that the codeposition of Cu, Bi, and Te occurs at around this potential as will be confirmed by EDS composition analysis in the following paragraph.

Typical thickness values of the binary and ternary films along with the elemental composition by EDS are presented in Table S1 of the SI. The codeposition of the elements in the ternary CuBiTe system is supported by the measured atomic composition of the thin films. From Figure 1b, it can be seen that the composition of films is changing with the addition of copper. Only small variation is observed in the “Te” composition, implying that the inclusion of Cu plays a little role in the Te content. However, after Cu1.0, there is a slight decrease in the Te content. In contrast, there is a drastic change in Bi composition in the presence of Cu in the ternary system. Since the formation of the CuBiTe ternary alloy produces a negative Gibbs free energy, it promotes the deposition of less noble metal [Cu = −0.09] at a high rate than the other components in the bath.^47^ Therefore, deposition of Cu is much higher than that of Bi, which leads to a high Cu:Bi ratio in the ternary alloys. It can be realized that a possible codeposition mechanism could be anomalous, which reduces the composition of Bi in the film when increasing the Cu concentration.^36,48^ Therefore, deposition of Bi inhibits in the presence of Cu as observed in the EDS data analysis. However, further investigations are needed to understand the anomalous codeposition phenomenon in the complicated ternary alloy system.

### 3.2 Morphology and Phase Analysis

**Figure 2:**
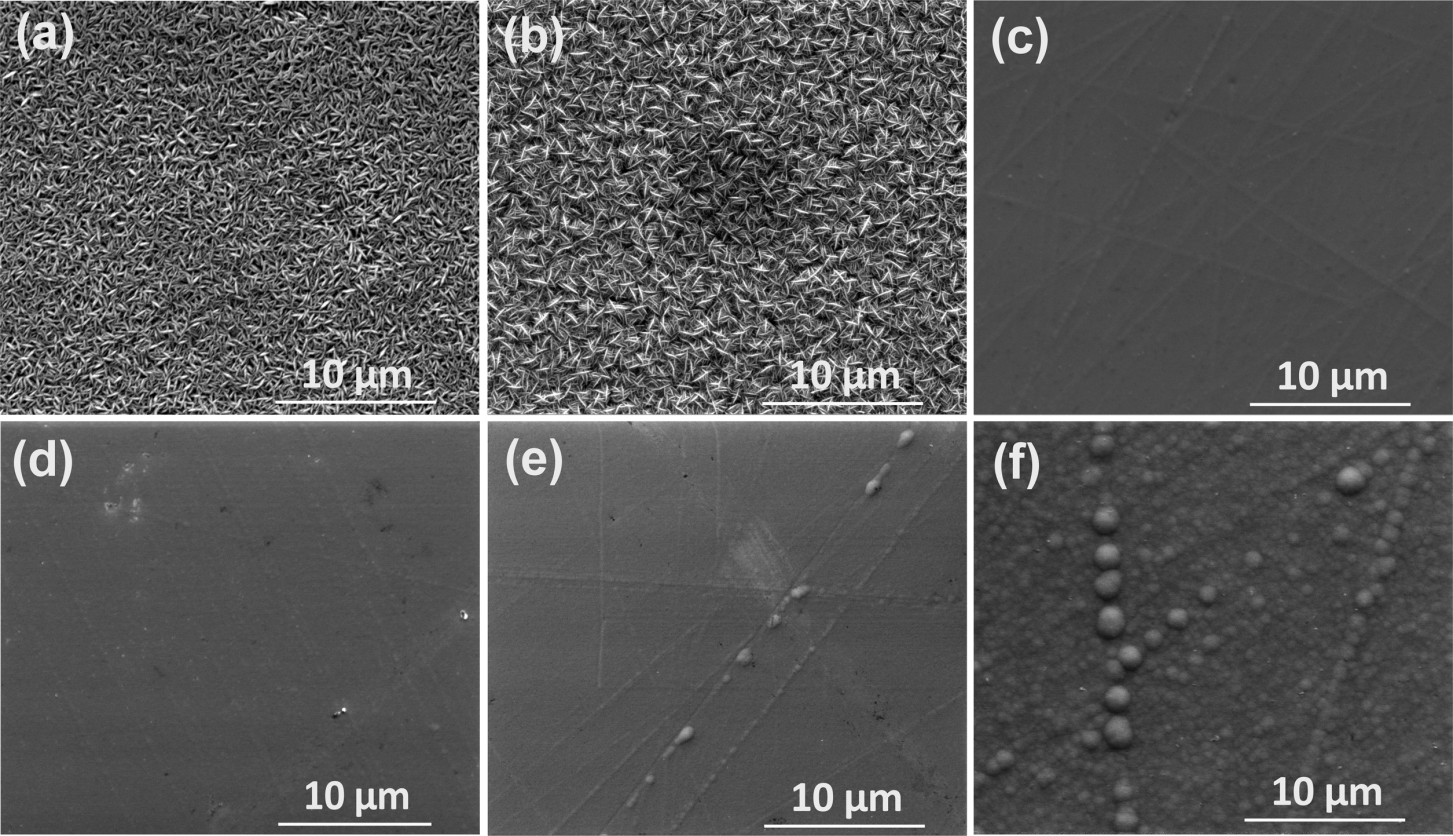
(a–f) SEM images of Bi_2_Te_3_ and Cu-added Bi_2_Te_3_ thin films: (a) Bi_2_Te_3_, (b) Cu0.5BiTe, (c) Cu1.0BiTe, (d) Cu1.5BiTe, (e) Cu2.0BiTe, and (f) Cu4.0BiTe.

**Figure 3:**
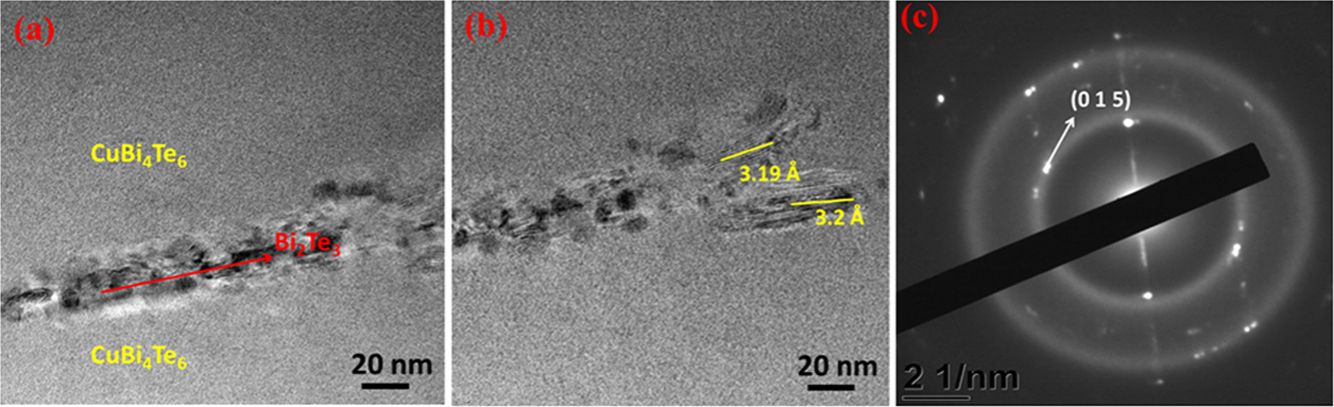
(a–c) HRTEM images of Cu1.0BiTe thin films and the corresponding SAED pattern.

Figure 2a–f shows the SEM images of electrodeposited Bi_2_Te_3_ and CuBiTe alloy thin films. The as-deposited Bi_2_Te_3_ binary alloy (Figure 2a) shows a wirelike morphology with uniform distribution of bismuth and tellurium. When 0.5 mM Cu is added, the deposited film morphology is changed to a microscale wirelike hierarchical surface with many branches. On further increasing the Cu concentration to ≥ 1 mM, the wirelike morphology disappears and an unevenly smooth film with precipitates is observed as shown in Figure 2c–f. From the TEM images, we can see only the amorphous structure without any crystalline features as depicted in Figure S3a–c. Interestingly, the HRTEM images shown in Figure 3a–c evidence the presence of nanocrystallites within the amorphous framework. The calculated d-spacing value (3.2 Å) and the corresponding plane (0 1 5) indicate that the crystallites are none other than Bi_2_Te_3_. The observed dot spots with diffused rings in the selected-area electron diffraction (SAED) pattern further support the presence of Bi_2_Te_3_ nanocrystals. Noticeably, at 4 mM Cu, no visible precipitates are found in the TEM analysis, and this shows the complete reformation of the amorphous CuBiTe alloy.

**Figure 4:**
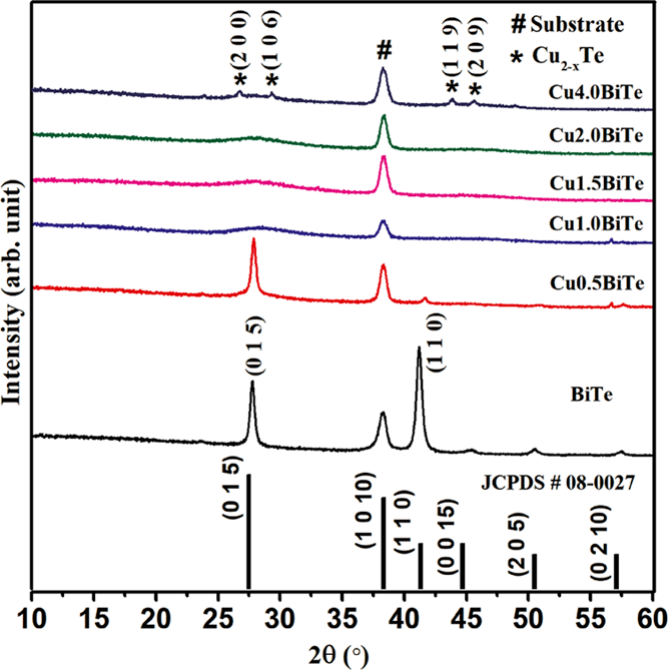
XRD patterns of as-deposited Bi_2_Te_3_ thin films with and without the addition of copper.

To further study the effect of Cu inclusion, X-ray diffraction is carried out on all of the samples. Figure 4 displays the typical XRD patterns of the as-deposited binary and ternary thin films. Diffraction peaks of (015) and (110) are found for the as-deposited Bi_2_Te_3_ sample, confirming film’s crystallinity. With the exception of the peaks from the substrate, all of the diffraction peaks can be indexed to rhombohedral Bi_2_Te_3_ (JCPDS file #08-0027; *a* = 4.386 Å, *c* = 30.497 Å).^49^ The crystalline Bi_2_Te_3_ is strongly oriented in the (110) direction as compared to the (015) direction, as shown in Figure 4.

The pattern exhibits different full widths at half-maximum of (110) and (105), indicating a larger average crystallite size in the (110) orientation and thus the growth of Bi_2_Te_3_ with multiple branches along the main direction with a hierarchical surface morphology.^50^ There is no evidence for any other phases in the XRD pattern for the binary system. However, in the ternary system, only the (015) orientation is observed, while the (110) peak is diminished at a lower concentration of Cu (0.5 mM), representing the downfall or degradation in the crystallinity of Bi_2_Te_3_ alloy thin films. When the Cu concentration is further increased to 1 mM, the material becomes completely amorphous with patterns manifesting an amorphous hump. This demonstrates a high degree of lattice distortion in the system, which leads to the crystal symmetry breakage. Thus, the addition of Cu beyond a certain concentration range can effectively reduce the crystallinity with the emergence of the amorphous state in CuBiTe alloy films. However, when the Cu concentration is high (4 mM), the phase segregation is visible with negligible intensity in the XRD pattern. All of the XRD peaks indexed as (200), (106), (109), and (209) are matched to the Cu_2–_*_x_*Te phase (JCPDS #10-0421) with a hexagonal structure.

### 3.3 Raman Analysis

**Figure 5:**
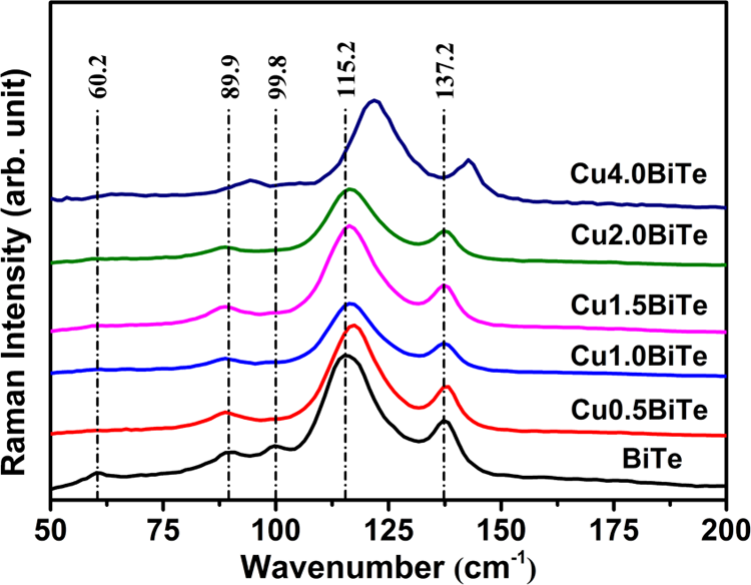
Raman spectra of pristine Bi_2_Te_3_ and CuBiTe thin films.

The Raman spectra of pristine Bi_2_Te_3_ and CuBiTe thin films are shown in Figure 5. The reproducibility of the Raman spectra is discussed in Figure S4 in the SI. It is well established that the primitive unit cell of Bi_2_Te_3_ contains five atoms in accordance with the chemical formula. Generally, bulk Bi_2_Te_3_ has 15 dynamical modes at q = 0, 3 of which are acoustic modes and 12 are optical modes. These 12 optical phonon modes are known to be 2 A_1g_, 2 E_g_, 2 E_u_, and 2 A_1u_. Among them, the A_1g_ and A_1u_ vibration modes are along the out-of-plane direction, whereas the E_g_ modes are along the in-plane direction.^51^ The deconvoluted Raman peaks are presented in Figure S5 in the SI. In Figure 5, for binary Bi_2_Te_3_, the optical phonon modes at 60.2 (^1^A_1g_) and 99.8 cm^–1^ (^2^E_g_) can be identified. However, discrepancies are observed when compared to the bulk samples.^49^ Here, the Eg_1_ mode (∼40 cm^–1^) is missing and the sample exhibits three additional bands at 89.9, 115.2, and 137.2 cm^–1^, which can be assigned to the respective E_1_, A_1_, and E_2_ active modes of Te-rich BiTe.^51^ These peaks may be originated from native defects including antisite defects (Bi_Te_^–^), structural defects (Bi_3_Te_4_^–^), and excess Te phase decomposed by a Raman laser, which leads to strong Te Raman features.^52^ The existence of native defects is associated with the stoichiometry of Bi atoms in Bi_2_Te_3._ The addition of Cu in the Bi_2_Te_3_ system shows similar Raman spectra to those of pristine Bi_2_Te_3_. However, variation in the relative peak intensity when Cu is added as well as a shift in the peak positions at the highest concentration of Cu is observed, which indicates that Cu is disturbing the Bi_2_Te_3_ layer structure by replacing Bi, as evident from the EDX analysis. It is worth mentioning that the Raman peaks are shifted to a higher wavenumber with more Cu incorporation, though it is not consistent. This may be due to the different occupation state of smaller Cu atoms in Bi_2_Te_3_ lattices at high concentration, where significant variation/change in the chemical bonds can happen and ultimately results in the structural disorder.^53^ Noticeably, the ^1^A_1g_ (60.2 cm^–1^) and ^2^E_g_ (99.8 cm^–1^) active modes disappear for CuBiTe films due to the formation of the ternary alloy, which may be due to the breaking of the crystal symmetry of Bi_2_Te_3_ through the addition of a high amount of Cu. Therefore, further crystallization of Bi_2_Te_3_ falls down with the increasing Cu concentration up to 1 mM samples and an amorphous phase is observed in the XRD pattern. The shift (>7 cm^–1^) in the Raman peak for the 4 mM Cu concentration sample further confirms the chemical composition change from CuBiTe to the formation of a Cu_2–_*_x_*Te solid solution.

### 3.4 XPS Analysis

**Figure 6:**
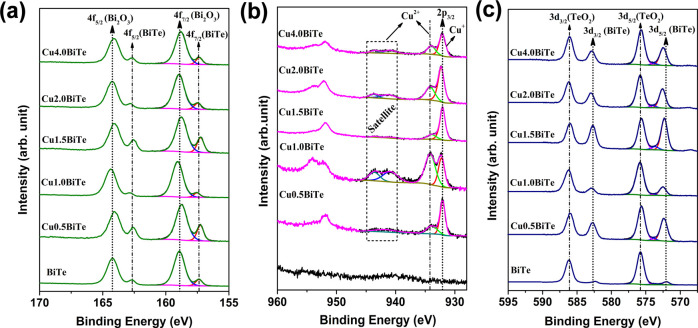
Core-level XPS signals of Bi 4f (a), Cu 2p (b), and Te 3d (c) for the as-deposited pure and Cu-added BiTe thin films at film surfaces.

X-ray photoelectron spectroscopy (XPS) could provide quantitative and direct analysis on the oxidation state of the elements and stoichiometry and allow the study of electron transfer by probing the chemical shift of the electronic structure of the elements involved. Figure 6a–c shows the core-level XPS signals of Bi, Te, and Cu for the as-deposited pure and Cu-added BiTe thin films. The survey spectrum of both binary and ternary systems shown in Figure S6 demonstrates that the samples are composed of bismuth, tellurium, copper, and a certain amount of oxygen. The presence of O1s peak in the spectra indicates surface oxidation, which generally takes place after the sample is exposed to the atmosphere.^54^ The core-level Bi 4f XPS spectra shown in Figure 6a show that the peaks are deconvoluted into two peaks at binding energies of 158.9 and 164.2 eV, which correspond to Bi 4f_7/2_ and Bi 4f_5/2_ spin–orbit splitting of Bi_2_O_3_.^55^ It can be clearly seen that the peaks at 157.3 and 162.6 eV are from Bi 4f, denoting the presence of Bi in a 3^+^ state. Moreover, the addition of Cu into the BiTe matrix results in a decrease in the intensity of Bi 4f peaks (Bi_2_O_3_), which slightly shift (0.2 eV) toward a low-energy regime. These measured shifts are quite low and within the energy resolution of the XPS instrument and thereby confirming that the addition of Cu does not affect the binding energies of the binary alloy at the surface.^56^ However, the existence of a high-intensity Bi_2_O_3_ peak can be resulted from the easy surface oxidation of binary and ternary bismuth chalcogenides upon exposing to atmosphere.^54^ As a consequence, it is essential for the films to be stored under an inert atmosphere to avoid surface oxidation. The Cu 2p XPS spectra for ternary systems are shown in Figure 6b. With the 19.7 eV spin–orbit separation, two distinct peaks positioned at 932.1 and 951.8 eV can be assigned to Cu 2p_3/2_ and Cu 2p_1/2_, respectively.^57^ Moreover, the prominent peak at 932.1 eV is related to Cu^+^ intercalation or diffusion into the interstitial position of the Bi_2_Te_3_ layered structure.^58^ The peaks of Cu 2p confirm the dual oxidation (Cu^+^/Cu^++^) states of Cu, with a different ratio. It is very hard to distinguish the two states of Cu in the XPS spectra. The prominent way to define the characteristic Cu^2+^ state is a satellite peak, and it should appear at ∼940 eV.^59^ In Figure 6b, Cu 2p spectra have the satellite peak in the 940–943 eV regime for all of the ternary alloys, indicating the multiple oxidation states of Cu. This may be attributed to either available 3d conduction states in CuBiTe, which allows for the taking on of multiple oxidation states, or atmospheric-exposure-triggered oxidation.^60^ It is worth mentioning that the 1 mM Cu-added Bi_2_Te_3_ system has the highest Cu^+^/Cu^++^ratio of 0.98, signifying that the two states of Cu have been equally distributed in the alloy. A further increase in Cu concentration shows the ratios of 1.25, 1.15, and 1.1 for 1.5, 2, and 4 mM copper concentrations, respectively.

Figure 6c depicts the core-level XPS spectra of Te 3d spin–orbit splitting exhibiting strong peaks at binding energies of 586.2 and 575.8 eV, which are in good agreement with Te 3d_3/2_ and Te 3d_5/2_ due to the surface oxide layer of TeO_2_. There are two peaks at binding energies of 572.0 and 582.5 eV, which can be attributed to actual Te 3d_5/2_ and Te 3d_3/2_ splits related to Bi–Te layers.^55^ The oxide layer results from air oxidation of the Bi_2_Te_3_ surface. The surface oxidation of this electrodeposited ternary CuBiTe is further supported by the XPS depth profile studies on the 1 mM Cu concentration sample. The depth profile confirms surface oxidation for few tens of nanometers and is shown in Figure S7. Subsequently, the overall XPS result confirms that the as-deposited films consist of Bi_2_Te_3_ and CuBiTe without any detectable residual secondary phases except for the Cu_2–_*_x_*Te solid solution at a higher Cu (4 mM) concentration.

### 3.5 Thermoelectric Studies

**Figure 7:**
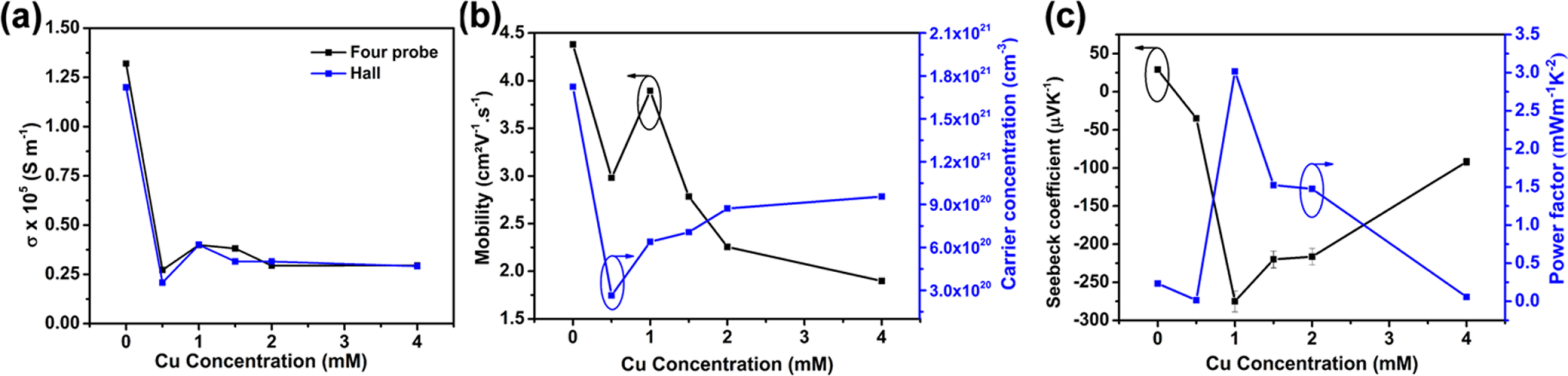
(a) Electrical conductivity of as-deposited pure Bi_2_Te_3_ and CuBiTe at different Cu concentrations measured by two conventional techniques. (b) Correlation between the Hall mobility and carrier concentration of the films with respect Cu concentration. (c) Measured Seebeck coefficient and calculated power factor variation of the film with Cu content.

To evaluate these electrodeposited bismuth telluride and copper bismuth telluride films as thermoelectric materials, their thermoelectric transport properties are investigated as a function of Cu concentration as shown in Figure 7. From Figure 7a, it can be seen that the room-temperature electrical conductivities (σ) are on the order of 10^5^ S m^–1^ for both binary and ternary alloys. For comparison, the electrical conductivity is measured using a conventional four-probe technique, which follows the same trend and is in agreement with the Hall data. However, to keep the consistency, we used the electrical conductivities determined by the Hall measurement for the thermoelectric analysis. While adding Cu into the Bi_2_Te_3_ binary system, the σ falls down from 1.2 × 10^5^ S m^–1^ to the (0.2–0.4) × 10^5^ S m^–1^ range. The high electrical conductivity of binary Bi_2_Te_3_ could be contributed by the desired (110) orientation and high Te content with reasonable crystallinity of the film.^61^ With the addition of Cu, there is a collapse of the crystal structure (Figure 2), which leads to the crystallinity loss and decreases the electrical conductivity significantly.^46^ This can be further explained through the variation in the carrier density and the mobility of the system due to the addition of Cu as obtained from the Hall measurements. The carrier mobility of a semiconductor is directly related to its electrical conductivity by the relation^12^
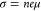
3where *n* is the carrier concentration (cm^–3^), *e* is the charge of an electron (1.602 × 10^–19^ C), and μ is the electron mobility (cm^2^ V^–1^ s^–1^). Figure 7b shows the change in carrier concentration (*n*) and the Hall mobility (μ_H_) as a function of Cu concentration. The as-deposited Bi_2_Te_3_ film shows a high p-type carrier concentration (1.7 × 10^21^ cm^–3^) with maximum mobility of 4.38 cm^2^ V^–1^ s^–1^. Clearly, by the addition of Cu into the films, a sudden fall in the mobility and carrier concentration can be observed for the film with 0.5 mM Cu. However, with a further increase in the Cu concentration in the films, the carrier concentration increases and a sudden increase in mobility for the sample with 1 mM Cu concentration is also observed. Upon a further increase in Cu concentration, a decrease in mobility is observed for the rest of the samples. It is noteworthy that the sample with 1 mM Cu concentration has optimum carrier concentration with higher mobility values compared to other samples with higher Cu concentrations, resulting in a higher electrical conductivity (3.99 × 10^4^ S m^–1^) of the sample. When the amount of Cu further increases, a certain amount of Cu replaces the Bi atom and starts to change the chemical composition by compensating holes, thereby decreasing the density of states near the Fermi level. Due to the low density of states, the corresponding carrier mobility is somewhat low for higher Cu concentration samples. Furthermore, the carrier type has been changed from a p-type as-deposited sample to n-type for 0.5 mM Cu, and its concentration decreases at a low Cu content and follows the increasing trend with increasing Cu concentration. At low Cu concentration (7.77 atom % for the 0.5 mM sample), the available p-type carriers are compensated by excess n-type carriers created due to Cu inclusion and n-type characteristics appear. More Cu in the BiTe system during codeposition acts as a donor, increasing the free electron density and thereby decreasing the hole concentration, which exists in the pristine Bi_2_Te_3_.^39^ By the addition of Cu, there may be a shift in the Fermi level from the upper edge of the valence band to the conduction band, which leads to the p-to-n crossover conductivity in CuBiTe at room temperature. The Fermi level can move further up and lies above the lower edge of the conduction band with increasing Cu concentration. Hence, the p-type carrier concentration reaches its minimum and the n-type carrier concentration rises accordingly.

Figure 7c displays the variation of Seebeck coefficient (*S*) and power factor as a function of Cu addition. As expected, Bi_2_Te_3_ exhibits a positive *S* value representing a p-type thermoelectric response of the material. With ∼8 atom % Cu into the BiTe system, the Seebeck coefficient becomes negative, suggesting that the majority carriers are electrons as Cu plays a donor role in the CuBiTe system. The room-temperature Seebeck coefficient varies accordingly with the carrier concentration as discussed above and can be related by the following Mott relationship:^62^
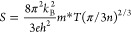
4where *S* is the Seebeck coefficient, *k*_B_ is the Boltzmann constant, *e* is the electronic charge, *h* is the Planck constant, m* is the density of state effective mass, *T* is the absolute temperature, and *n* is the carrier concentration. With a low Cu content, the *S* value is low, which then rapidly increases for 1 mM Cu and then falls down for higher Cu (1.5–2 mM) concentrations. This observation can be related to the carrier concentration, since there is often an optimal value of charge carrier density at which the Seebeck coefficient is maximum.^62^ A higher carrier concentration would result in a lower Seebeck coefficient, which is also supported by our electrical conductivity measurements.^63^ Typically, the absolute Seebeck coefficient increases abruptly from −34.9 to −275 μV K^–1^ for 1 mM Cu and starts to decrease again to −220, −216.4, and −133.6 μV K^–1^ for 1.5, 2.0, and 4.0 mM Cu concentrations, respectively. It is worth noting that the 1.5 and 2 mM Cu concentrations exhibit nearly similar Seebeck coefficients, which agrees well with their carrier concentration and electrical conductivity. This may cause a greater increase in carrier density and result in a reduction of Seebeck coefficient. The abrupt increase of Seebeck coefficient at room temperature for 1 mM Cu can be explained by the optimal value of carrier concentration, increase in local density of states near the Fermi level, and the structural change.^58,59^

The power factor is an important thermoelectric parameter, which can be calculated using the Seebeck coefficient and electrical conductivity (PF = *S*^2^σ).^62^Figure 7c shows the power factors for the electrodeposited Bi_2_Te_3_ films with different amounts of Cu at room temperature. Interestingly, a maximum power factor of 3.02 mW m^–1^ K^–2^ is obtained for the 1 mM Cu-added Bi_2_Te_3_ thin film owing to its good electrical conductivity of 3.99 × 10^4^ S m^–1^ and a maximum Seebeck coefficient of −275 μV K^–1^. The samples that showed similar Seebeck coefficients (−220 and −216.42 μV K^–1^) exhibited a bit lower power factors of 1.52 and 1.47 mW m^–1^ K^–2^, respectively. The estimated Seebeck and power factor values are much higher than those of the various electrochemically deposited thermoelectric thin films reported so far and are presented in Table S2 in the SI. Furthermore, these values are well correlated to the reported power factor of the Cu*_x_*Bi_2_Te_2.7_Se_0.3_ (PF = 3.15 mW m^–1^ K^–2^) bulk sample.^64^ A considerably low power factor was obtained when compared to the result (5.3 mW m^–1^ K^–2^) reported for Cu*_x_*Bi_2_(Te_0.9_Se_0.1_)_3_, which may be due to the limited donor contribution from the Cu-induced amorphous framework.^65,66^ This indicates that the addition of Cu greatly tunes the power factor and the 1 mM Cu sample could be a better choice for further improvement of power factor and, ultimately, the figure of merit (*zT*).

This extraordinary power factor of our electrochemically codeposited CuBiTe ternary alloy is due to the pronounced enhancement of its Seebeck coefficient and the nominal electrical conductivity due to the formation of ternary CuBiTe systems, which reorganize an original layered structure to a new amorphous framework. Therefore, this approach will give a new strategy to further enhance the desirable power factor of Bi_2_Te_3_ thin films by reducing their crystallinity at near room temperature. However, caution should be needed for high-temperature thermoelectric applications. A further study related to the structural stability of these materials in a medium–high temperature regime is in progress.

### 3.6 Structure–Property Relation

Bi_2_Te_3_ doped with transition metals is a well-known high-performance thermoelectric material with an enhanced figure of merit (ZT ∼ 1) at elevated temperatures. Particularly, the addition of Cu into the Bi_2_Te_3_ layered structure is being studied extensively to improve the thermoelectric properties of the system in the realm of thin films.^38,42,67^ Studies showed that the Cu addition could influence the electrical conductivity, Hall coefficient, and thermal power due to the dopant-induced structural defects.^38^ Although few research studies focused on the structure and position of Cu within the Bi_2_Te_3_ layers, no studies had been done on electrodeposited CuBiTe materials explaining the destruction of the crystal symmetry and enhancement of thermoelectric properties. With the proposed Cu substitution at the Bi site, due to the valence of the Cu^++^, it could act as an acceptor doping. The observed negative Seebeck coefficient and n-type carriers can be correlated with the possibility of incorporation of Cu into the compound, which increases the electrical conductivity to a further extent. Originally, the as-deposited Bi_2_Te_3_ exhibits a high concentration of holes; hence, it should require a high amount of Cu to crossover the p-to-n-type conductivity. From the composition analysis, it is evident that >7 atom % of Cu is present in the BiTe system, which shows dominant n-type carriers for all of the CuBiTe ternary alloys.

From XRD analysis, it is clearly visible that there is a strong distortion in the crystal structure of CuBiTe samples. The crystallinity of Bi_2_Te_3_ falls down with increased Cu concentration, where samples deposited from 1 to 2 mM Cu concentrations clearly evidence the amorphous nature. A deeper study of this amorphous phase by STEM reveals Bi_2_Te_3_ nanocrystals embedded in the system for samples fabricated from 1 mM Cu concentration. Here, the two possible mechanisms for Cu addition are as follows: (1) Cu sits in the van der Waals gap and chemical exfoliation happens during Bi_2_Te_3_ quintuple formation or (2) Cu replaces Bi and interacts with the Te (2) layer. Both the mechanisms can distort the crystallinity of the CuBiTe ternary system and leads to amorphization with embedded Bi_2_Te_3_ nanocrystals. Recently, it has been observed that the addition of an excess amount of Cu atoms into Bi_2_Te_3_ favors the formation of Cu clusters.^60^ Since our bath contains excess Te^2–^ and Cu^2+^ ions, it should readily form the Cu_2–_*_x_*Te secondary phase during electrodeposition. At a high Cu concentration, the emergence of Cu_2–_*_x_*Te phase segregation is noticeable (Figure 4), which is due to the fact that the excess Cu reacts with Te to form the secondary phase within the host matrix. However, there is no direct evidence for the presence of Cu_2–_*_x_*Te at low Cu (1 mM) concentrations. Therefore, it can be suggested that Cu_2–_*_x_*Te does not play any major role in the observed thermoelectric transport properties. The anomalous variation in the electrical conductivity and carrier concentration may be due to the Cu ion substitution, which acts as a donor impurity, thus increasing the amount of carrier. As evidenced from HRTEM, the few layers of crystalline Bi_2_Te_3_ present within the amorphous framework could form a charge transport channel in the CuBiTe amorphous matrix. These nanocrystalline Bi_2_Te_3_ layers may play a key role in abruptly increasing the carrier mobility in 1 mM Cu-added BiTe films.^68^ Additionally, these ultrathin Bi_2_Te_3_ nanocrystals in the amorphous matrix may act as an energy barrier for carriers and a phonon scattering center.^69^ With a further increase of Cu concentration, the Cu_2–_*_x_*Te secondary phase is formed, which limits the carrier mobility by the formation of new phases and thus low electrical transport properties are observed. Therefore, further investigation on stability and temperature-dependent thermoelectric studies are required to stabilize the materials for their application in future micro-thermoelectric devices, and the work is in progress.

## 4 Conclusions

In summary, we have studied the thermoelectric characteristic performance of Cu-doped BiTe thin films fabricated through the electrodeposition technique. To our surprise, the crystallinity of the Bi_2_Te_3_ binary system collapsed with the addition of >7 atom % Cu, which transformed the material into an amorphous phase. Interestingly, when the Cu level was 13 atom % in BiTe, the carrier mobility increased abruptly and delivered the highest power factor of 3.02 mW m^–1^ K^–2^. The observed superior carrier concentration and electrical conductivity and excellent power factor values demonstrate a new strategy in emerging high-efficiency room-temperature thermoelectric materials by the simple electrodeposition technique that CuBiTe can be a promising n-type material for high-efficiency thermoelectric device applications. Further stabilization of the material for application over a wide temperature range could make CuBiTe a superior n-type candidate for near-room-temperature thermoelectric power generation applications.

## Supporting Information Available

Seebeck coefficient measurement setup description, film thickness and composition table, TEM images, Raman spectra measured at different regions of the film surface, deconvoluted Raman spectra, XPS survey spectrum, core-level XPS spectra of CuBiTe thin films at different etching times, and thermoelectric performance comparison table (PDF)
