# Supplementary material for: Amorphous Framework in Electrodeposited CuBiTe Thermoelectric Thin Films with High Room-Temperature Performance
Source: ACS Appl Electron Mater. 2021 Apr 7;3(4):1794–803. doi: 10.1021/acsaelm.1c00063 (PMC8824429; doi:10.1021/acsaelm.1c00063)
Supplement: Supplementary file 1 — el1c00063_si_001.pdf [file el1c00063_si_001.pdf]

Supporting Information

Amorphous framework in electrodeposited CuBiTe thermoelectric Thin-films with High Room Temperature performance

N. Padmanathan[a,b#], Swatchith Lal[a#], Devendraprakash Gautam[a] and Kafil M. Razeeb[a]*

[a]Micro-Nano Systems Centre, Tyndall National Institute, University College Cork, Dyke Parade, Lee Maltings, Cork T12 R5CP, Ireland.

[b]Department of Physics, Karpagam Academy of Higher Education, Coimbatore, Tamilnadu, India - 641021

*Corresponding Author: Dr. Kafil M. Razeeb (kafil.mahmood@tyndall.ie)

#These authors contributed equally to this work.

The Seebeck coefficient measurements

The schematic of the Seebeck measurement setup is shown in Figure S6. The Seebeck coefficient of all samples was evaluated in the in-plane configuration by establishing a temperature gradient (ΔT) along the length of the sample through commercially available Peltier modules at the two ends of the sample as shown in Figure S1.

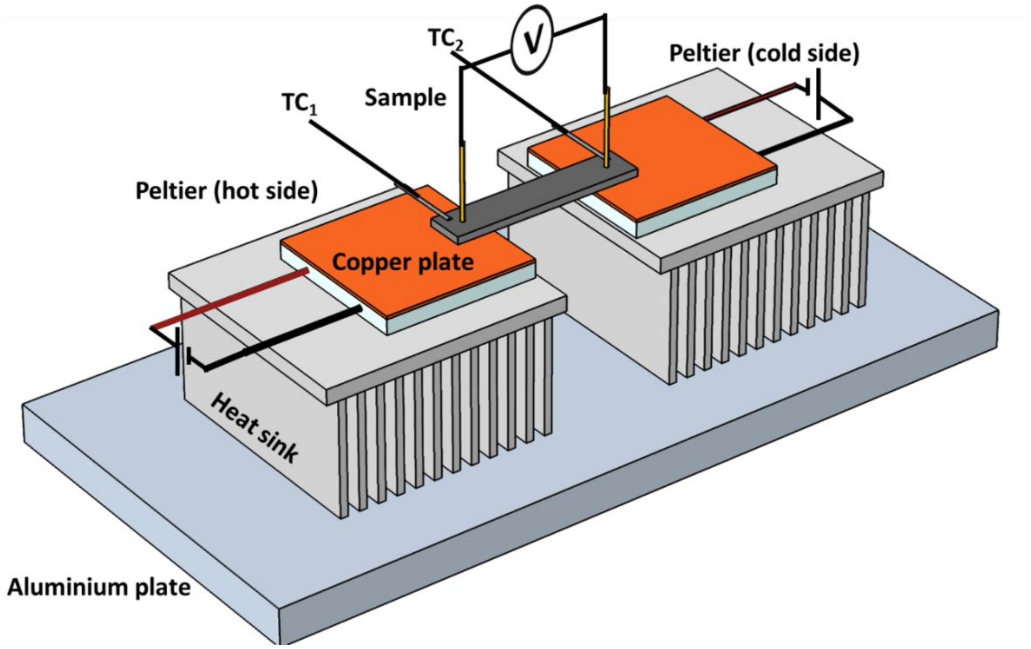

Figure S1. Schematic of the Seebeck coefficient measurement system of thin films. [ACS Appl. Energy Mater. 2020, 3, 4, 3262–3268][1]. Copyright [2020] American Chemical Society

Two separate thermocouples (Type K) situated at the ends of the sample recorded the temperatures (TC_1 and TC_2). The thermocouples were in direct contact with the sample ensuring minimal thermal losses. The temperature gradient between both the ends was measured using a thermocouple reader. The temperature of the cold was kept at a fixed temperature for e.g. at room temperature (298 K), and a temperature gradient of 2 to 10 K was created by increasing the temperature of the Peltier module on the hot side.

The sample was placed on the Peltier module and to ensure proper thermal contact of the sample with the Peltier modules, boron nitride-based commercial thermal interface materials was used between the sample and the Peltier module. The voltage readings were taken by a spring-loaded flat and spherical ended gold microprobe ensuring a good electrical contact with the thermoelectric film without cracking, stressing and damaging the films.

Similar setups are well known and have been reported in the literature for near room temperature Seebeck coefficient measurement of the thermoelectric materials [2-4].

The thermovoltage was measured in the range of $\Delta T \rightarrow$ 2-10 K using a high impedance voltmeter. Two different runs of increasing and decreasing ΔT were performed to ensure the measurements obtained are stable and correct. In run-1 the ΔT was increased from 2 K to 10 K with an interval of 2 K for every thermovoltage measurement. The measured data were recorded only when both the temperature gradient and the thermovoltage depict stable value thereby minimizing the measurement errors. In run-2 the ΔT was decreased back from 10 K to 2 K. All the obtained thermovoltage data are plotted against ΔT. The thermovoltage dependence on the temperature gradient depicts a linear behaviour. The Seebeck coefficient is determined from the slope of the curve.

Table 1. The average film thickness and the elemental composition of the electrodeposited films.

**Sample**	**Average Film Thickness**	**At.% - Bi**	**At.% - Te**	**At.% - Cu**
BiTe	1.2 µm	36.04±2	63.96±2	0
Cu0.5BiTe	1.6 µm	29.11±0.1	63.12±0.1	7.77±0.1
Cu1.0BiTe	1.85 µm	23.4±0.5	63.19±0.5	13.4±0.5
Cu1.5BiTe	1.85 µm	17.11±1	60.9±1	21.99±1
Cu2.0BiTe	1.85 µm	16.24±0.5	59.95±0.5	24.57±0.5
Cu4.0BiTe	1.6 µm	9.29±0.9	52.49±0.9	38.22±0.9

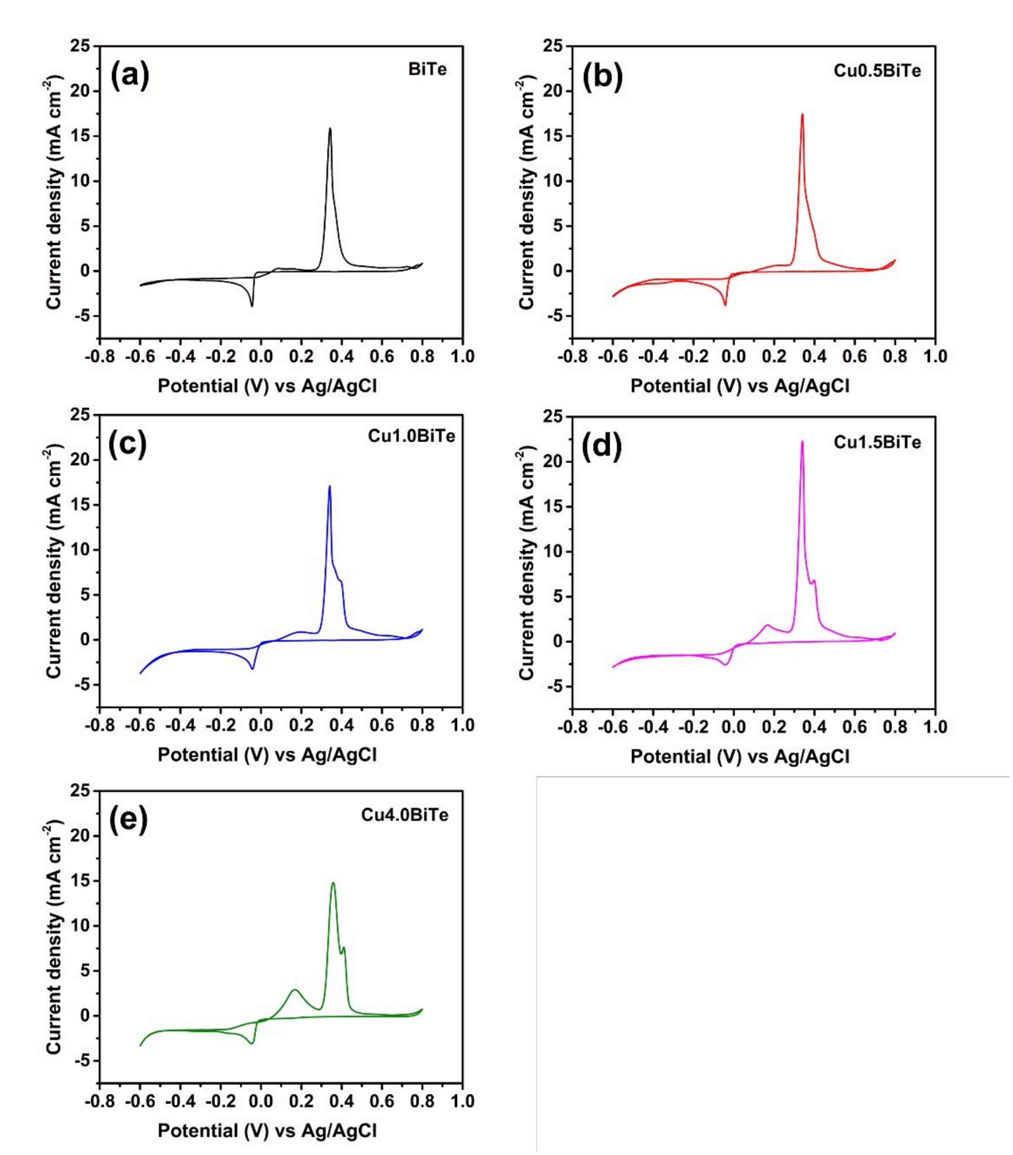

Figure S2. The CV curves observed at 10 mVs^{-1} for (a) pure BiTe, (b) Cu0.5BiTe, (c) Cu1.0BiTe, (d) Cu1.5BiTe and (e) Cu4BiTe.

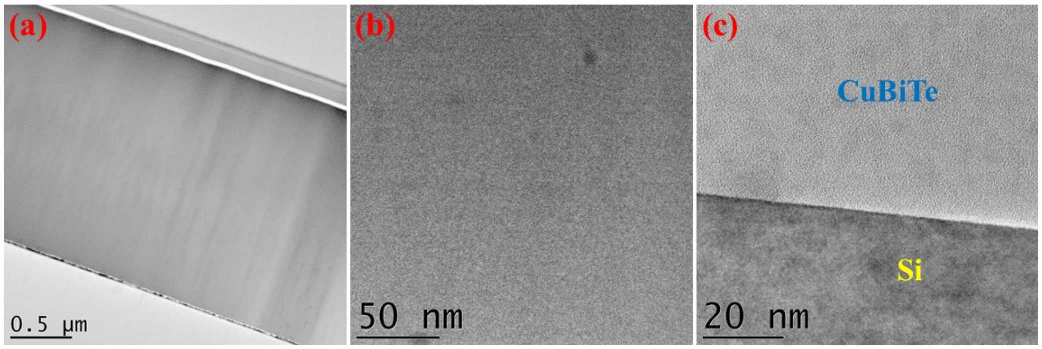

Figure S3. (a-c) TEM images of Cu1.0BiTe thin films at different resolutions.

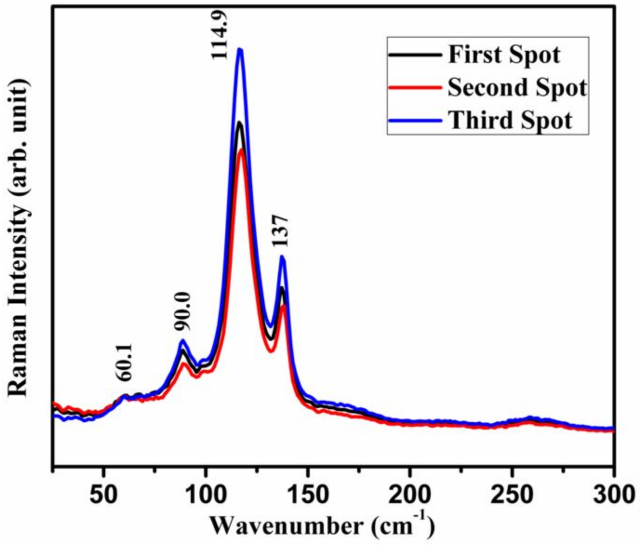

Figure S4. Raman spectra of CuBiTe thin film with 1 mM Cu measured at different points on the film surface.

In order to confirm the reproducibility, the spectra were taken at three different spots on the film. We observed the difference in the intensity between different spots as shown in Fig. S3. The intensity variation could result from the varying thickness of the film over the surface of the substrate.

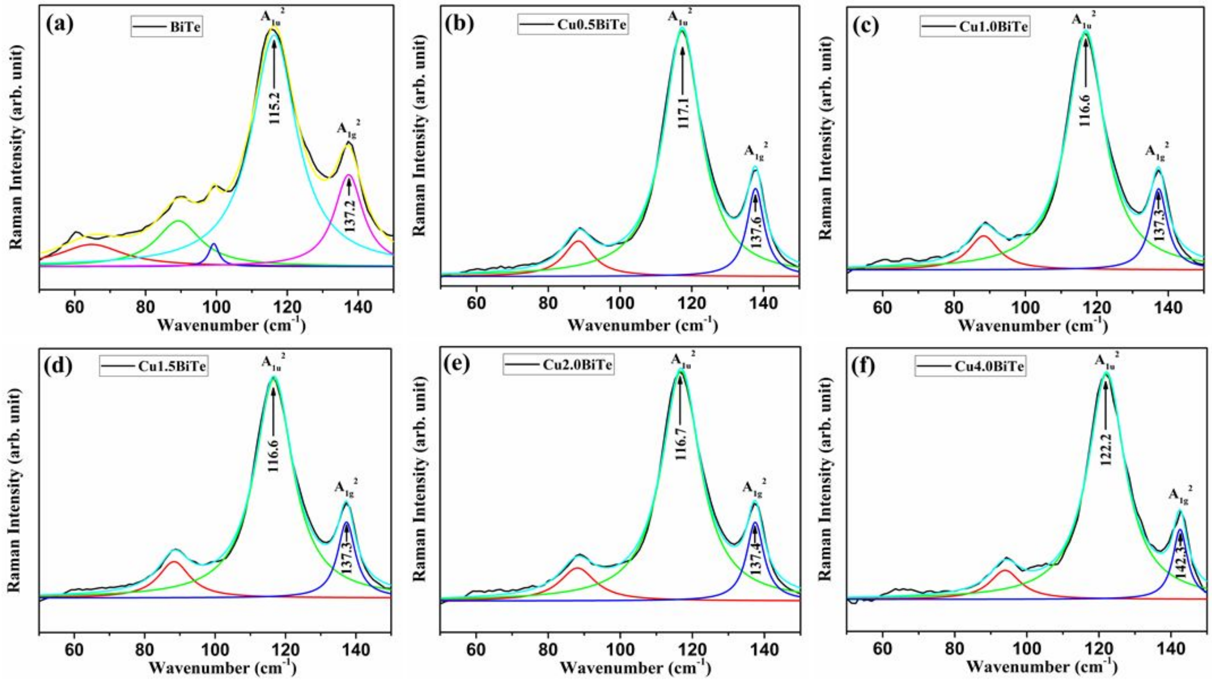

Figure S5. (a-f) De-convoluted Raman A_{1u}^2 and A_{1g}^2 for both pure and Cu added Bi_2Te_3 thin films.

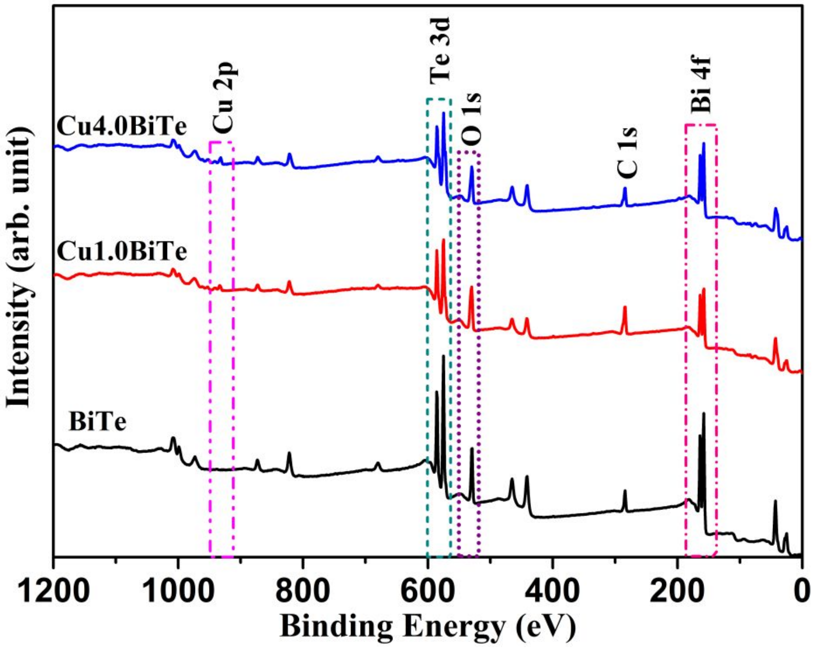

Figure S6. XPS survey spectrum of pure Bi_2Te_3 and CuBiTe with 1 mM and 4 mM Cu addition.

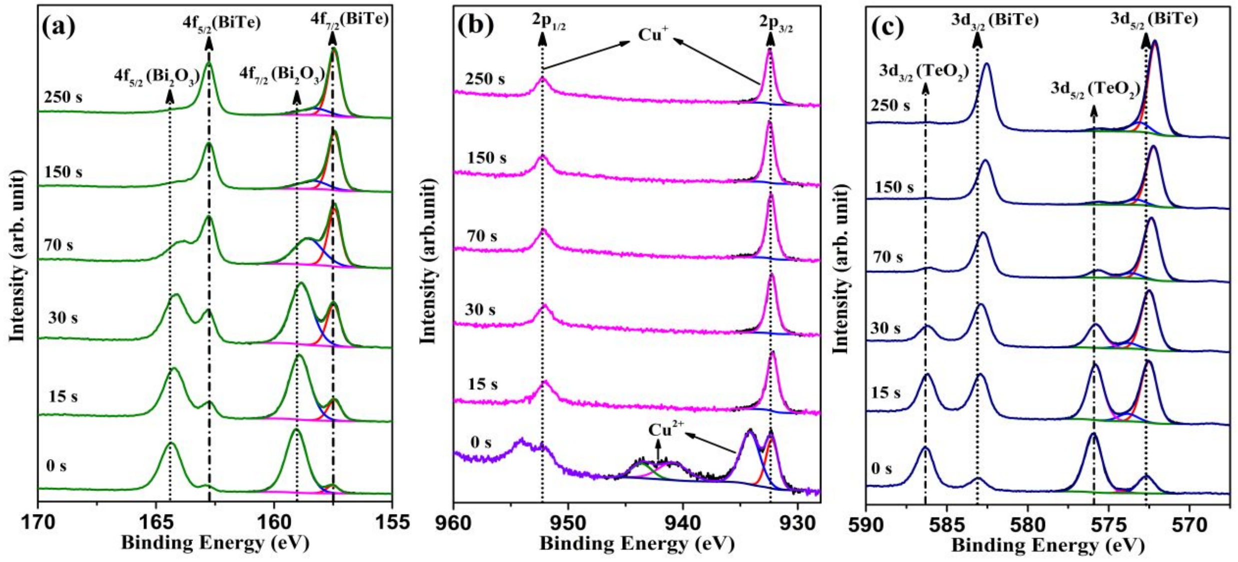

Figure S7. The core level XPS signals of Bi4f (a), Cu2p (b) and Te3d (c) for Cu1.0 BiTe thin films at different etching time.

Table 2: Thermoelectric properties of room-temperature thermoelectric thin-films along with their best power-factor values are given in the table.

***ECD – electrochemical deposition**

References	Material	n-/p-type	Deposition Technology	Seebeck coefficient (μVK^{-1})	Electrical conductivity (Sm^{-1})	Power-factor ($mWm^{-1}K^{-2}$)
**This work**	**CuBiTe**	**n-type**	**ECD**	**-257**	**3.9×10^4**	**3.02**
1	Cu dope Te	n-type	ECD	-227	1.0×10^5	5.62
5	$Bi_2Te_{3-x}Se_x$	n-type	ECD	-105.9	1.7×10^5	1.91
6	Bi_2Te_3	n-type	ECD	-200	4.0×10^4	1.60
7	$Bi_{2.17}Te_{2.83}$	n-type	ECD	-146	6.9×10^4	1.47
8	Sb_2Te_3	p-type	ECD	*~117	1.0×10^5	1.36
9	$(Bi_xSb_{1-x})_2Te_3$	p-type	ECD	182	4.0×10^4	1.32
	$Bi_2(Te_xSe_{1-x})_3$	n-type	ECD	-130	4.8×10^4	0.82
10	Bi_2Te_3	n-type	ECD	-100	*~7.1×10^4	0.74
11	Bi_2Te_3	n-type	ECD	-130	4.0×10^4	0.68

References:

(1) Lal, S.; Razeeb, K. M.; Gautam, D. Enhanced thermoelectric properties of electrodeposited Cu-doped Te films. *ACS Applied Energy Materials* **2020,** *3* (4), 3262-3268.

(2) Borup, K. A.; de Boor, J.; Wang, H.; Drymiotis, F.; Gascoin, F.; Shi, X.; Chen, L.; Fedorov, M. I.; Müller, E.; Iversen, B. B.; Snyder, G. J. Measuring thermoelectric transport properties of materials. *Energy & Environmental Science* **2015,** *8* (2), 423-435, DOI: 10.1039/C4EE01320D.

(3) Loureiro, J.; Neves, N.; Barros, R.; Mateus, T.; Santos, R.; Filonovich, S.; Reparaz, S.; Sotomayor-Torres, C. M.; Wyczisk, F.; Divay, L.; Martins, R.; Ferreira, I. Transparent aluminium zinc oxide thin films with enhanced thermoelectric properties. *Journal of Materials Chemistry A* **2014,** *2* (18), 6649-6655, DOI: 10.1039/C3TA15052F.

(4) Iwanaga, S.; Toberer, E. S.; LaLonde, A.; Snyder, G. J. A high temperature apparatus for measurement of the Seebeck coefficient. *Review of Scientific Instruments* **2011,** *82* (6), 063905, DOI: 10.1063/1.3601358.

(5) Kim, J.; Lee, K. H.; Kim, S. W.; Lim, J.-H. Potential-current co-adjusted pulse electrodeposition for highly (110)-oriented Bi2Te3-xSex films. *Journal of Alloys and Compounds* **2019,** *787*, 767-771, DOI: https://doi.org/10.1016/j.jallcom.2019.01.301.

(6) Lei, C.; Burton, M. R.; Nandhakumar, I. S. Facile production of thermoelectric bismuth telluride thick films in the presence of polyvinyl alcohol. *Physical Chemistry Chemical Physics* **2016,** *18* (21), 14164-14167, DOI: 10.1039/C6CP02360F.

(7) Na, J.; Kim, Y.; Park, T.; Park, C.; Kim, E. Preparation of Bismuth Telluride Films with High Thermoelectric Power Factor. *ACS Applied Materials & Interfaces* **2016,** *8* (47), 32392-32400, DOI: 10.1021/acsami.6b10188.

(8) Hatsuta, N.; Takemori, D.; Takashiri, M. Effect of thermal annealing on the structural and thermoelectric properties of electrodeposited antimony telluride thin films. *Journal of Alloys and Compounds* **2016,** *685*, 147-152.

(9) Schumacher, C.; Reinsberg, K. G.; Rostek, R.; Akinsinde, L.; Baessler, S.; Zastrow, S.; Rampelberg, G.; Woias, P.; Detavernier, C.; Broekaert, J. A. C.; Bachmann, J.; Nielsch, K. Optimizations of Pulsed Plated p and n-type Bi2Te3-Based Ternary Compounds by Annealing in Different Ambient Atmospheres. *Advanced Energy Materials* **2013,** *3* (1), 95-104, DOI: 10.1002/aenm.201200417.

(10) Heo, P.; Hagiwara, K.; Ichino, R.; Okido, M. Electrodeposition and Thermoelectric Characterization of Bi[sub 2]Te[sub 3]. *Journal of The Electrochemical Society* **2006,** *153* (4), C213, DOI: 10.1149/1.2168378.
(11) Li, S.; Soliman, H. M. A.; Zhou, J.; Toprak, M. S.; Muhammed, M.; Platzek, D.; Ziolkowski, P.; Müller, E. Effects of Annealing and Doping on Nanostructured Bismuth Telluride Thick Films. *Chemistry of Materials* **2008,** *20* (13), 4403-4410, DOI: 10.1021/cm800696h.
